# Rapid detection of glycopeptide-resistant enterococci: impact on decision-making and costs

**DOI:** 10.1186/2047-2994-2-30

**Published:** 2013-11-04

**Authors:** Gabriel Birgand, Raymond Ruimy, Michael Schwarzinger, Isabelle Lolom, Gisèle Bendjelloul, Nadira Houhou, Laurence Armand-Lefevre, Antoine Andremont, Yazdan Yazdanpanah, Jean-Christophe Lucet

**Affiliations:** 1 IAME, UMR 1137, Univ Paris Diderot, Sorbonne Paris Cité, F-75018 Paris, France; 2 IAME, UMR 1137, INSERM, F-75018 Paris, France; 3 AP-HP, Hôpital Bichat, Infection Control Unit, F-75018 Paris, France; 4 Bacteriology laboratory, Bichat-Claude Bernard Hospital, Paris, France; 5 Virology laboratory, Bichat-Claude Bernard Hospital, Paris, France; 6 AP-HP, Hôpital Bichat, Service de Maladies Infectieuses et Tropicales, F-75018 Paris, France

**Keywords:** Screening, Glycopeptide, Resistant, Enterococci, Cost, Decision-making, Search and isolate

## Abstract

**Background:**

According to French national recommendations, the detection of a patient colonized with glycopeptide-resistant enterococci (GRE) leads to interruption of new admissions and transfer of contact patients (CPs) to another unit or healthcare facility, with weekly screening of CPs.

**Findings:**

We evaluated the medical and economic impact of a pragmatic adaptation of national guidelines associated with a real-time PCR (RTP) (Cepheid Xpert™ *van*A/*van*B) as part of the strategy for controlling GRE spread in two medical wards. Screening was previously performed using chromogenic selective medium (CSM). Turn around time (TAT), costs of tests and cost of missed patient days were prospectively collected. In February 2012, the identification of GRE in one patient in the diabetology ward led to the screening of 31 CPs using CSM; one secondary case was identified in a CP already transferred to the Nephrology ward. Awaiting the results of SCM (median TAT, 70.5 h), 41 potential patient days were missed, due to interruption of admissions. The overall cost (screening tests + missing patient.days) was estimated at 14, 302.20 €. The secondary case led to screening of 22 CPs in the Nephrology ward using RTP. Because of a short median TAT of 4.6 h, we did not interrupt admissions and patients’ transfers. Among 22 CPs, 19 (86%) were negative for *van*A, 2 were positive for *van*B and 3 had invalid results needing CSM. The overall cost of the strategy was estimated at 870.40 € (cost of screening tests only), without missing patient days.

**Conclusion:**

The rapid PCR test for *van*A-positive GRE detection both allowed rapid decision about the best infection control strategy and prevented loss of income due to discontinuation of patient transfers and admissions.

## Findings

Glycopeptide-resistant in enterococci (GRE) have risen rapidly in recent years, posing a particular threat in healthcare facilities (HCF) [1]. Several countries have issued national guidelines for controlling their spread [2,3]. French recommendations consist of strict contact precautions for colonized patients in screening and isolation of contact patients (i.e. sharing the nursing staff with colonized patients), with neither transfers of patients nor admissions to the involved ward until three negative weekly screening tests [4]. The percentage of GRE (mostly *van*A genotypes) among *E. faecium* bloodstream infections in France declined from a 3.2% peak in 2006 to a plateau at about 1% afterwards [5]. However, this strategy is burdensome and limits the use of hospital services, resulting in both missed opportunities for patients and decreased income for HCF.

The cornerstone of those control measures is the early recognition of GRE colonized patients by rapid and accurate screening tests. The Xpert™ system (Cepheid, Sunnyvale, CA) is a one-step PCR assay providing results generally in less than 1 h with a high negative predictive value (NPV) for GRE detection in rectal specimens. However, the carriage of *van*B gene by other organisms than *E. faecium* gives a weak positive predictive value to the test [6]. The purpose of this study was to evaluate the medical and economic impact of the Cepheid Xpert™ *van*A/*van*B real-time PCR assay as part of a strategy for controlling GRE spread in two medical wards.

## Patients and methods

### Study design

The present study is a description and evaluation of two control measures. The first was applied for an index case in a diabetology ward and included a screening based on culture technique. The second used a PCR assay and was applied for a secondary case hospitalized in a Nephrology ward.

### Local recommendation

We adapted French national recommendations based on the pragmatic following rules: (i) if no new GRE case was identified by initial cross-sectional screening of contact patients, patient transfer to other wards or HCFs were allowed, as well as the admission of new patients. Contact patients transferred to another ward were to be placed in single room with contact precautions in the downstream ward until the result of two subsequent screening tests was available. (ii) If another GRE-positive case was identified by cross-sectional screening, national guidelines were to be followed scrupulously.

### Microbiological tests

Two simultaneous culture techniques were used: (i) One rectal swab inoculated in enrichment broth (AES VRE) incubated 24 hours, and then subcultured onto the chromogenic medium (Oxoid Brillance VRE) incubated aerobically at 37°C and read after 24 and 48 h; (ii) a second swab was directly plated on the same type of medium. *Enterococcus faecium* were identified using a mass spectrometry assay (MALDI-TOF-MS system). Strains were suspected as GRE in case of minimum inhibitory concentrations (MICs) >8 mg/L for vancomycin and/or teicoplanin using E-test strips (BioRad). Vancomycin-resistance genotypes were identified using a DNA strip assay (GenoType Enterococcus; Hain Lifescience GmbH).

For the molecular diagnosis, Xpert *van*A PCR assay was performed by the manufacturer’s instructions (Cepheid) using rectal swabs. Considering the high negative predictive value of the test, the patient was considered at low risk to be colonized if the result was negative for the *van*A and *van*B genes. Otherwise, conventional culturing was performed to confirm or disprove the presence of GRE. Cultures were also performed in case of amplification inhibitors in the sample.

### Time and cost analysis

Time required for each step of the microbiological analysis was collected prospectively. The cost of screening was computed based on the use of material resources needed and personnel costs on the basis of the hourly salary of a senior staff member.

We estimated the costs attributable to the decreased occupancy by multiplying the number of missed patient-days (difference between admission capacity and the number of admitted patients when a GRE-positive patient was identified) by the mean cost billed per hospital day (total amount billed in 2011 based on French Diagnosis-Related Groups divided by the number of patient-days in 2011) [7].

## Results

### Investigation around the first case: local recommendation and culture method

On 2 February, 2012, a 68-year-old man was admitted to the diabetology ward for the management of complicated type-2 diabetes.

On 27 February, an *E. faecium* strain highly resistant to vancomycin and teicoplanin was cultured from a wound in the right foot PCR confirmed the presence of the *van*A gene. The diabetology ward has 32 beds in 16 double rooms. All 31 patients hospitalized in the ward were considered contact patients of the first case. At this time, the Xpert™ *van*A/*van*B PCR had been recently introduced in our laboratory and was used on an exceptional basis to screen the two patients who had shared the room of the first case patient, one of whom was PCR-positive. The investigation of this secondary case will be described in the corresponding paragraph.

On 28 February, rectal swabs were obtained from the 31 contact patients of the first case and cultured according to standard techniques. Transfers to other units or HCFs and admissions were stopped pending the results of the rectal-swab cultures. However, 17 of the 31 contact patients were sampled and discharged home over the next two days.

On 29 February, the two colonized patients (the initial case and the secondary case identified from the same room by PCR) and 13 contact patients were cohorted in a separate area of the ward and cared for by dedicated staff to prevent cross transmissions.

As shown in Table 1, the median turnaround times (TAT) for culture techniques was 70.5 hours. Of the 31 screened patients, one was found colonized with GRE corresponding to the secondary case previously identified by PCR (see above). None of the 17 patients discharged home was finally found to be colonized.

**Table 1:** **Description of results, time and cost of microbiological analysis during two phases of the investigation of two cases of glycopeptide-resistant *Enterococci***

	**Investigation of the first case in the diabetology unit**	**Investigation of a secondary case in the nephrology unit**
	**(n=31 patients)**	**(n=22 patients)**
**Test Results,** n (%)		
Cepheid Xpert™ *van*A/*van*B assay:		
Negative PCR	1 (3)^a^	17 (77)
Positive *van*A	1 (3)^a^	0 (0)
Positive *van*B and culture showing susceptible strain	-	2 (9)
PCR invalid then negative culture	-	2 (9)
PCR invalid then culture showing susceptible stain	-	1 (5)
Culture after enrichment using chromogenic medium:		
Negative culture	26 (84)	-
Culture positive for GRE strain^b^	1 (3)^a^	-
Culture positive for susceptible *Enterococcus*^c^	4 (13)	-
**Turn-around time** (hours, median [Q1-Q3])		
From sampling, to sample reception	2.6 (1.7-2.6)	2.8 (1.1 – 3.8)
From sample reception, to inoculation or preparation	2.3 (2.2– 2.4)	1.3 (0.5 – 2.3)
From inoculation or preparation, to results	65.5 (65.5– 65.5)	1 (0.9-1.1)
From sample reception, to results	67.8 (68.4 – 67.9)	6.22 (3.7 – 8.2)
From sampling, to results	70.5 (69.4 – 70.5)	4.6 (4.0 – 18.9)
Maximal time to obtain all results	70.5	90.0
**Cost of microbiological analysis (€)**		
Cepheid Xpert™ *van*A/*van*B assay:		
Cost of 1 cartridge	-	35.60
Cost of 1 test	-	37.30
Culture with chromogenic medium and enrichment		
Cost of a negative culture	4.80	-
Cost of a doubtful culture^c^	13.40	-
Cost of a doubtful culture^d^	23.50	-
Cost of a positive culture^b^	117.80	-
Total cost of microbiological testing	333.50	870.40
**Loss of income**		
Cost per weighted case per day in 2011^e^	340.70	426.00
*Scenario 1:* Implementation of local guidelines		
Patient-days lost ^f^	41	0
Estimated loss of income (€)	13,968.70	0
*Scenario 2:* Implementation of national guidelines		
Patient-days lost^f^	250	0
Estimated loss of income (€)	85,175.00	0
**Overall loss of income (€)**	13,968.70 to 85,175.00	0
**Overall cost of the strategy (€)**	14,302.20 to 86,175.50^g^	870.40 to 2,611.20^g^

PCR, polymerase chain reaction; GRE, Glycopeptide-Resistant Enterococci ; Q1, First quartile; Q3, Third quartile.

^a^Real-time PCR assay performed on an exceptional basis to screen the two patients who shared the room of the first patient in the diabetology ward.

^b^Positive culture with identification of *E. faecium* by mass spectrometry assay, MICs >8 mg/L for vancomycin and/or teicoplanin on E-test strips, antibiotic susceptibility testing by disk diffusion in solid media for clinical purpose, and detection of the vancomycin resistance genotype by DNA strip assay.

^c^Positive culture with identification of *Enterococcus faecium* by mass spectrometry assay with MICs ≤8 mg/L for vancomycin and/or teicoplanin on E-test strips.

^d^Positive culture with identification of *E. faecium* by mass spectrometry assay, MICs >8 mg/L for vancomycin and/or teicoplanin on E-test strips, and antibiotic susceptibility testing by disk diffusion in solid media for clinical purpose.

^e^Estimated costs of inpatient care based on reimbursement rates of the diagnosis-related group. In France, the diagnosis-related group price is calculated by multiplying standard amounts for operating and capital expenses found in yearly surveys by a national “weight” associated with the DRG for each hospitalisation. The weighting takes in account variations due to geographic area and atypical observations.

^f^Number of missed patient-days due to the interruption of patients’ transfers and admissions.

^g^Costs estimation assuming the strict implementation of French national guidelines with three weekly screening of patients.

On 1 March, new admissions were allowed in a different area of the ward. Two additional weekly screening of contact patients performed using the same culture method identified no additional cases.

This local GRE control protocol resulted in a 72-hour period without admissions or transfers, with 41 missed patient-days and €13,968 of lost income (Table 1). Following the national guidelines would have resulted in 15 days without admissions or transfers, with 250 patient-days of loss of activity and €85,175 of lost income. The cost of microbiological testing using the culture method was €333.50. The global estimated costs were therefore €14,302 and €86,175 with the local and national guidelines, respectively.

### Investigation around the secondary case: local recommendation and PCR assay

As described previously, a secondary case was rapidly identified using the Xpert™ *van*A/*van*B PCR. This patient stayed in the nephrology ward from 1 January to 20 February, 2012. The nephrology ward has 28 beds with 12 double and 4 single rooms. On 28 February, the 22 patients hospitalized during the same period and still present in the nephrology ward were considered to be contact patients and were screened for GRE by rectal swabbing. We decided to use the GeneXpert™ test for this purpose and, given the TAT with this test, to continue transfers and admissions as usual unless another GRE-positive patient was identified. The median time to results was 4.6 hours after sampling (Table 1). However, because only a four-site GeneXpert™ system was available, the results for all 22 patients were obtained 9.5 hours after sampling, and decision about the GRE-control strategy was therefore made at the end of the day. None of the contact patients had *van*A-positive strains. Consequently, transfers and admissions were continued. The overall cost of PCR testing for *van*A-positive GRE was €870.40.

## Discussion

This observational study showed that using the rapid PCR test for *van*A-positive GRE detection both allowed rapid decisions about the best infection control strategy and prevented loss of income due to discontinuation of patient transfers and admissions. To our knowledge, our study is the first to precisely evaluate the impact of rapid PCR for decision making in a context of GRE outbreak.

French authorities have issued strict guidelines for controlling highly-resistant bacteria such as GRE and carbapenemase-producing enterobacteriaceae [4]. These guidelines are based on the assumption that successfully controlling emerging pathogens will ultimately be cost-effective [8,9]. However, these strict guidelines result in significant loss of healthcare activity, as patient transfers and admissions are stopped pending results of repeated tests to identify secondary cases are available. Our locally-adapted national guidelines are based on a pragmatic attitude. In a context of fortuitous GRE identification, the epidemic potential is assessed using the first cross sectional screening. Duration of restrictions in patient transfers and admissions depend on the rapidity to obtain screening results. We calculated that a single GRE case handled according to local recommendations resulted in a loss of €14,302 for the diabetology ward, assuming the use of standard culture methods. Had the national recommendations been followed, the maximal loss would have been €86,175. However, cohort nursing of the colonized and contact patients would have resulted in lower costs. In both situations, rapid PCR screening was estimated to result in substantial cost savings.

Rapid GRE detection using the Xpert™ *van*A/*van*B PCR has several advantages. First, the time to results is approximately one hour from arrival of the specimen at the laboratory. However, simultaneous screening of numerous patients requires a platform equipped with several modules. Second, the GeneXpert™ system has a high NPV for *van*A and *van*B [10]. However, other normal inhabitants of the gut flora may exhibit *van*B genes, resulting in a lower NPV and requiring conventional cultures in case of *van*B positive PVR assay.

The main limitations of our study are the observational design and the small number of cases and units in the cluster.

In conclusion, a rapid real-time PCR assay contributes to decision-making regarding GRE control measures and resulted in substantial cost savings. Additional studies on a larger scale and with control groups are required to confirm our results.

## Abbreviations

GRE: Glycopeptide-resistant enterococci; WS: Weekly screening; CP: Contact patients; PCR: Polymerase chain reaction; RTP: Real-time PCR; CSM: Chromogenic selective medium; TAT: Turn around time; HCF: Healthcare facilities; NPV: Negative predictive value.

## Competing interests

The authors declare that they have no competing interests.

## Authors’ contributions

GB, II, GiBe and JCL participated to the data collection; II, RR, LAL, NH, and AA carried out the microbiologic analysis; JCL, MS, YY and AA provided writing assistance. All authors read and approved the final manuscript.

## Authors’ information

GB (PharmD, MPH) is a fellow in infection control. II is a BS involved in the infection control activity of the Bichat-Claude Bernard hospital, GiBe is an infection control nurse. RR (MD, PhD) and LAL (PharmD, PhD) are both bacteriologists at the Bichat-Claude Bernard hospital and specialised in molecular biology. NH is virologist at the Bichat-Claude Bernard hospital. YY (MD, PhD) is head of the infectious disease department and the ATIP-Avenir, Inserm U995 team. MS (MD, PhD) is a specialised in health economic. JCL (MD, PhD) is head of the infection control unit at the Bichat-Claude Bernard hospital.
